# MicroRNAs 10a and 10b Regulate the Expression of Human Platelet Glycoprotein Ibα for Normal Megakaryopoiesis

**DOI:** 10.3390/ijms17111873

**Published:** 2016-11-09

**Authors:** Zuping Zhang, Yali Ran, Tanner S. Shaw, Yuandong Peng

**Affiliations:** 1 School of Basic Medicine, Central South University, Changsha 410013, China; zhangzuping@csu.edu.cn; 2 Cardiovascular Research Section, Department of Medicine, Baylor College of Medicine, Houston, TX 77030, USA; Ran_yali@163.com (Y.R.); tshaw@bcm.edu (T.S.S.)

**Keywords:** microRNA, gene regulation, platelet glycoprotein Ibα

## Abstract

MicroRNAs are a class of small non-coding RNAs that bind to the three prime untranslated region (3′-UTR) of target mRNAs. They cause a cleavage or an inhibition of the translation of target mRNAs, thus regulating gene expression. Here, we employed three prediction tools to search for potential miRNA target sites in the 3′-UTR of the human platelet glycoprotein (*GP*) *1BA* gene. A luciferase reporter assay shows that miR-10a and -10b sites are functional. When miR-10a or -10b mimics were transfected into the GP Ibβ/GP IX-expressing cells, along with a DNA construct harboring both the coding and 3′-UTR sequences of the human *GP1BA* gene, we found that they inhibit the transient expression of GP Ibα on the cell surface. When the miR-10a or -10b mimics were introduced into murine progenitor cells, upon megakaryocyte differentiation, we found that GP Ibα mRNA expression was markedly reduced, suggesting that a miRNA-induced mRNA degradation is at work. Thus, our study identifies GP Ibα as a novel target of miR-10a and -10b, suggesting that a drastic reduction in the levels of miR-10a and -10b in the late stage of megakaryopoiesis is required to allow the expression of human GP Ibα and the formation of the GP Ib-IX-V complex.

## 1. Introduction

Platelet glycoprotein (GP) Ibα is exclusively expressed on the surface of megakaryocytes and platelets. It associates with platelet GP Ibβ, GP IX and GP V, forming one of the major protein complexes on the platelet surface. Normal function of the GP Ib-IX-V complex is required for platelet attachment, aggregation and activation under high shear flow conditions. Lack of or dysfunction in GP Ibα causes Bernard-Soulier syndrome (BSS), a hereditary bleeding disorder that is characterized by low platelet counts, giant platelets and a severe bleeding tendency [1].

In addition to its hemostatic role, GP Ibα can promote endomitosis and maturation of megakaryocytes, a process that is essential for the production of platelets [2,3]. In vitro investigation by culturing human CD34^+^ cells in the presence of a mixture of megakaryocyte differentiation-inducing cytokines has demonstrated that GP Ibα starts to appear in the middle stage of megakaryopoiesis, after which expression drastically increases to reach a maxima in the late stage of megakaryopoiesis [3]. Without GP Ibα, immature megakaryocytes accumulate in the murine bone marrow [4]. Even though it remains largely unknown how GP Ibα regulates megakaryocyte maturation, a number of investigations have suggested that extracellular binding to von Willebrand factor (vWf) [5,6] and intracellular association with either the membrane skeleton through actin binding protein 280 [7] or with 14-3-3ζ/phosphoinositide 3-kinase may be the mechanism [2]. Along these lines, little effort has been put forward to elucidate the mechanisms by which the expression of GP Ibα and other subunits in the GP Ib-IX-V complex is regulated. In the past several decades, there has only been one study that attempted to explore the regulatory elements for GP Ibα expression. Even though two transcriptional factors, *GATA* (GATA binding protein) and *Ets* (E26 transformation-specific sequence family transcriptional factor), were identified in that study as regulators of GP Ibα expression [8], it does not explain why GP IIb and GP Ib subunits (e.g., GP Ibα and GP V) appear at different stages of megkaryopoiesis [3], because both *GATAs* and *Ets* play similar roles in the transcription of these molecules [8,9,10,11]. Thus, it is possible that additional/specific regulatory mechanisms exist that drive the temporal expression of various megakaryocytic genes during the course of megakaryocyte maturation.

MicroRNAs are a class of small non-coding RNAs (~22 nt). They bind to the three prime untranslated region (3′-UTR) of target mRNAs, leading to a cleavage or an inhibition of the translation of target mRNAs [12]. As a post-transcriptional inhibitor, miRNAs play critical roles in the development of many cell lineages, one of which is megakaryocytes [13,14]. In vitro differentiation of the human CD34^+^ cells into megakaryocytes is accompanied by a down-regulation of numerous miRNAs and a simultaneous up-regulation of their target genes [15,16,17]. For instance, the transcriptional factor *MAFB* (V-maf musculoaponeurotic fibrosarcoma oncogene homolog B) is up-regulated during megakaryocyte differentiation upon a significant reduction of miR-130a [15]; miR-155 is also down-regulated which is accompanied by an increase in the expression of *Ets-1* and *Meis1* (Meis homeobox 1), two transcriptional factors that are important for megakaryocyte development [18]. In both cases, the down-regulation of these inhibitory miRNAs correlates well with the progression of megakaryocyte differentiation, suggesting that miRNAs indeed play important roles in this process.

We employed three prediction tools to search for potential miRNA target sites in the 3′-UTR of the human *GP1BA* gene, and results demonstrated that a number of miRNAs could potentially bind to this region. Subsequent measurement in a luciferase reporter assay shows that only the sites for miR-10a and -10b are functional. When we transfected miR-10a or -10b mimics into either the GP Ibβ/GP IX-expressing cells along with a DNA construct harboring both the coding and 3′-UTR sequences of the human *GP1BA* gene, or murine lineage negative cells prior to megakaryocytic differentiation, we found that miR-10a and -10b mimics inhibit the GP Ibα mRNA expression and transient expression of GP Ibα protein on the cell surface, as compared to the negative control miRNA or other miRNA mimics tested. Thus, our data demonstrate that GP Ibα is a novel regulatory target of miR-10a and -10b, and suggest that a reduction of the miR-10a and -10b levels is essential for the normal progression of the late stage of megakaryopoiesis by promoting a sufficient expression of GP Ibα and subsequent formation of the GP Ib-IX-V complex on the surface of megakaryocytes and platelets.

## 2. Results

### 2.1. Web-Based Prediction of miRNAs Targeting the Human GP Ibα mRNA

**Figure 1:**
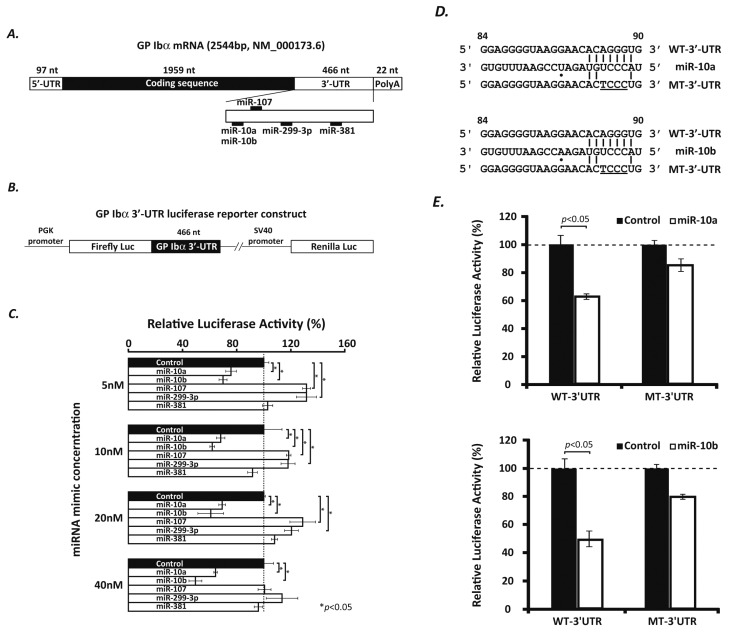
miR-10a and -10b target the 3′-UTR of the human *GP1BA* gene. (**A**) Schematic view of the 2054 nt (nucleotides) mRNA transcript of the human *GP1BA* gene illustrating the location of the coding sequence and the predicted binding sites for miR-10a, -10b, -107, -299-3p and -381 in the 3′-UTR; (**B**) Schematic view of the wild type reporter construct (WT-3′-UTR) that harbors a 466 nt region of the wild type human *GP1BA* 3′-UTR inserted downstream of a firefly luciferase coding sequence. Because the renilla luciferase gene is independently transcribed, it serves as an internal control in the luciferase assay; (**C**) Hela cells were co-transfected with 0.2 µg WT-3′-UTR reporter plasmid DNAs along with a series of concentrations (5, 10, 20 and 40 nM) of miRNA mimics (miR-10a, -10b, -107, -299-3p, and -381). Only the miR-10a and -10b mimics can significantly (* *p* < 0.05) reduce the firefly luciferase activity in a dose-dependent manner. Mean activities of firefly and renilla luciferases were measured 40 h after transfection and presented as ratios of firefly to renilla luciferase activity. Standard errors (±SEM) were calculated from three independent experiments in triplicate (*n* = 3); (**D**) Base pairing of miR-10a and -10b with their predicted target sites in the 3′-UTR of the human *GP1BA* gene. The mutations introduced to the seed sequence of a WT-3′-UTR are underlined; miR-10a and -10b differ in only one nucleotide (dotted); (**E**) The inhibitory effects of miR-10a and -10b mimics (40 nM) on firefly luciferase activities were greatly inhibited when the mutant reporter constructs (MT-3′-UTR) were used. The Student *t* test and analysis of variants were used to assess differences. *p* < 0.05 is considered as significant.

It has recently been appreciated that GP Ibα is important for the maturation of megakaryocytes [4]. Since its cloning in the late 1980s, however, very little has been discovered about the mechanism through which the expression of GP Ibα is regulated during megakaryocyte maturation. In recent years, miRNAs have been demonstrated to be important regulators of the development of many cell lineages, including megakaryocytes. The expression levels of a number of miRNAs vary in order to regulate the expression of their target genes during normal megakaryopoiesis. In the GP Ib-IX-V complex, GP Ibα expression is drastically increased in the late stage of megakaryocyte maturation, a temporal expression pattern that inspired us to hypothesize that GP Ibα may be regulated by miRNAs. To explore which miRNAs may be involved, we employed three web-based bioinformatics tools (TargetScan, miRanda and PITA) to analyze the 3′-UTR of the human *GP1BA* gene. A total of 173 miRNAs were predicted to bind to the 3′-UTR of the human *GP1BA* gene by TargetScan, seven of which were also returned by miRanda (miR-10a, -10b, -107, -153, -299-3p, -300 and -381). However, PITA prediction showed that only the first five of these seven miRNAs are potentially capable of targeting the human *GP1BA* gene (miR-10a, -10b, -107, -153, and -299-3p). Furthermore, by performing a literature review, we found that miR-10a, miR-10b and miR-107 were previously reported to be involved in the regulation of hematopoietic gene expression [15,19], and miR-299-3p is predicted to target the 3′-UTRs of both human and mouse *GP1BA* gene. Therefore, we chose to assess four miRNAs (miR-10a, miR-10b, miR-107, and miR-299-3p) and investigate if they can target the 3′-UTR and regulate the expression of human GP Ibα (Figure 1A). In addition, we still included miR-381 in our assay to evaluate the efficacy of our prediction approach as well as to ensure the specificity of the four miRNAs of interest.

### 2.2. miRNA 10a and 10b Regulate Human GP Ibα Expression

We made reporter constructs containing the wild type *GP1BA* 3′-UTR (466 nucleotides, nt) inserted downstream of a firefly luciferase coding sequence. A renilla luciferase gene, driven by independent transcriptional elements in the same construct, was used as an internal control (Figure 1B). After we co-transfected this reporter construct into HeLa cells along with various concentrations of miRNA mimics (ranging from 5 to 40 nM), only the cells co-transfected with the miR-10a or -10b mimics showed a significant reduction in their firefly luciferase activities (Figure 1C). In contrast, the miR-381 mimic showed little, if any, inhibitory effect. To further evaluate the specificity, we mutated the seed sequences of the putative miR-10a and -10b targeting sites in the *GP1BA* 3′-UTR (Figure 1D, underlined), and tested if miR-10a and -10b mimics could inhibit the activity of the firefly luciferase in the mutant construct as compared to the cells transfected with the wild-type constructs. Interestingly, we found that upon mutation the strong inhibitory effect we observed initially was abolished through use of the mutant constructs (Figure 1E), indicating that the predicted target sites of miR-10a and -10b in the 3′-UTR of the human *GP1BA* gene are functional.

**Figure 2:**
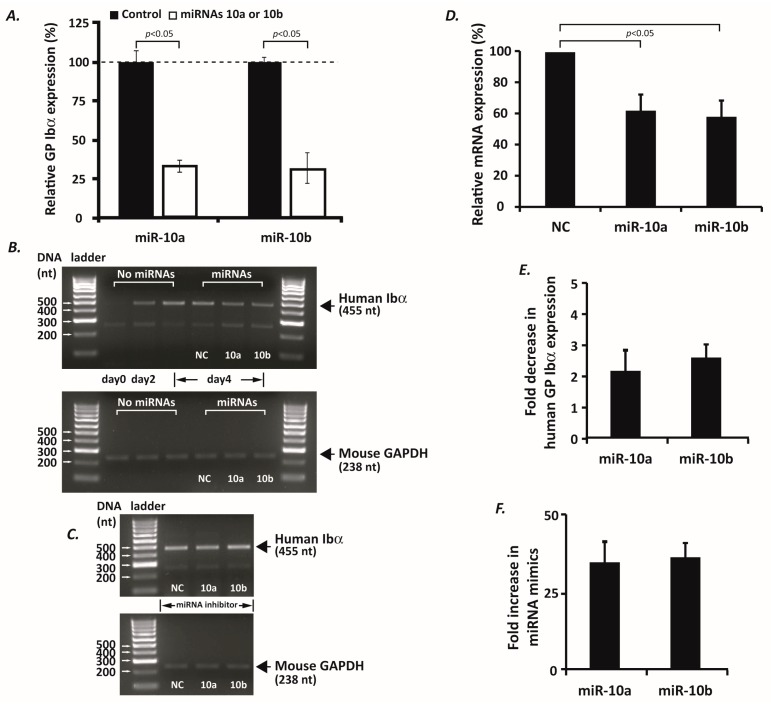
miR-10a and -10b inhibit the expression of human GP Ibα. Either miR-10a or -10b mimics (80 nM) were co-transfected with 0.5 µg of pDX-hGP Ibα plasmid DNAs, a human GP Ibα expression construct containing both coding and 3′-UTR sequences of the human *GP1BA* gene, into β/IX-expressing Chinese Hamster Ovary (CHO) cells. Two days after transfection, the transient expression levels of GP Ibα in these cells were measured by flow cytometry with a human GP Ibα-specific mouse monoclonal antibody and a fluorescein isothiocyanate (FITC)-labeled anti-mouse secondary antibody. Mean fluorescence intensities of the miR-10a/b mimic-transfected cells were normalized to that of the negative control miRNA mimic-transfected cells (**A**); Standard errors (±SEM) were calculated from three independent experiments. A half million murine lineage negative cells were transfected with 80 nM of miR-10a or -10b or negative control (NC) miRNA mimics, and then treated with a mixture of megakaryocyte differentiation-inducing cytokines as described in the Materials and Methods. In the 4-day period after transfection and megakaryocyte differentiation, the human GP Ibα mRNA expression levels were examined either by reverse transcription polymerase chain reaction (RT-PCR) (**B**) followed by densitometry analysis using Image J software (version 1.48) (**D**), or by quantitative PCR (**E**). Neither miR-10a nor -10b inhibitors increase the expression levels of human GP Ibα mRNA at day 2 (**C**). In addition, the expression levels of miRNA mimics transfected into the cells at day 4 were quantified by qPCR as well (**F**). The Student *t* test and analysis of variance were used to assess differences. *p* < 0.05 is considered as significant.

**Figure 3:**
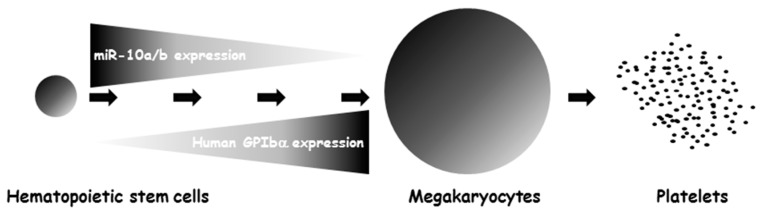
A schematic model illustrating how a drastic reduction in miR-10a and -10b levels allows sufficient expression of human GP Ibα and therein the GP Ib-IX-V complex in the late stage of megakaryocyte development. Because miR-10a and -10b do not introduce any mutations to the GP Ibα coding sequence and so do not alter the amino acid composition in the mature GP Ibα protein, it is unlikely that miR-10a and miR-10b can alter GP Ib-IX-V complex formation. However, sustaining high levels of miR-10a and miR-10b will cause degradation of GP Ibα mRNA, resulting in low amounts of GP Ibα protein and therein decreased expression levels of the GP Ib-IX-V complexes on the surface of the platelets. This will ultimately inhibit megakaryopoiesis and platelet production.

To further demonstrate that *GP1BA* is a target gene of miR-10a and -10b, we transfected the β/IX-expressing Chinese Hamster Ovary (CHO) cells with a mixture of the human GP Ibα expression vector and miR-10a/b or negative control miRNA mimics, to examine if GP Ibα expression changes at the protein level. After staining the cells with a GP Ibα specific antibody and analyzing the cell surface expression of GP Ibα by flow cytometry, we found that both miRNA mimics significantly reduce the protein levels of GP Ibα to ~30% of the average level in the negative control miRNA mimic-transfected cells (Figure 2A). Considering the fact that miR-10a and -10b differ in only one nucleotide (Figure 1D, dotted) and have an identical seed sequence, our data demonstrated that miR-10a and -10b can negatively regulate the expression of the *GP1BA* gene through the base pairing of their seed sequences to the complementary target site in the 3′-UTR of the human *GP1BA* gene. Interestingly, we also employed the same bioinformatics approach to analyze the 3'-UTRs of other GP Ib-IX-V subunit genes, i.e., GP Ibβ, GP IX and GP V, and found that, of the proteins forming the GP Ib-IX-V complex, miR-10a and -10b specifically regulate the human *GP1BA* gene only. Furthermore, in order to investigate the physiological relevance of our finding, we isolated the murine lineage negative cells from a well-established transgenic mouse line which only expresses human GP Ibα. This mouse was generated by an integration of an entire cassette of the human *GP1BA* gene into the mouse genome with removal of the endogenous murine *GP1BA* gene by genetic manipulation [20,21]. Prior to in vitro megakaryocytic differentiation by megakaryocyte differentiation-inducing cytokines (e.g., thrombopoietin and Interleukin-3), we transfected these progenitors with the various miRNA mimics of interest. As shown in Figure 2B, upon megakaryocytic differentiation, these cells progressively express increasing amounts of human GP Ibα mRNA, the level of which was decreased by ~40% (densitometry analysis, Figure 2D) or 2–3-fold (qPCR quantification, Figure 2E) 4 days after the introduction of exogenous miR-10a or -10b. We also quantified the levels of miR-10a and -10b mimics within the progenitor cells at day 4, and found the levels of transfected miR-10a and -10b mimics were increased by approximately 30-fold (Figure 2F), a ratio comparable to the degree of reduction of these two miRNAs in the late stage of human megakaryopoiesis (~12–50-fold) [15]. In contrast, the negative control miRNA mimic-transfected cells express equivalent levels of the human GP Ibα mRNA as compared to the non-transfected cells. In addition, we also tested miR-10a or -10b inhibitors in this megakaryocyte differentiation system; we observed neither an early expression of GP Ibα mRNA at day 1 nor a significant increase of GP Ibα mRNA level at day 2 (Figure 2C). One possible reason for this observation is that GP Ibα mRNA expression requires transcription factors which do not express with a sufficient amount in the early stage of megakaryopoiesis. Nevertheless, our data demonstrate that miR-10a and -10b can repress human *GP1BA* gene expression through miRNA-mediated mRNA degradation. To the best of our knowledge, miR-10a and -10b are the first two miRNAs that have been identified and experimentally validated for human *GP1BA* gene regulation.

## 3. Discussion

In the human genome, miR-10a is located upstream of the *HoxB4* (Homeobox B4) gene within the *HOXB* (Homeobox B) gene cluster of chromosome 17q21 and its expression corresponds to that of *HoxB4*. During megakaryopoiesis, HoxB4 up-regulates molecules, such as Scl-1 [22] and Fli-1 [23], or down-regulates *c-Myb* (avian myeloblastosis virus oncogene cellular homolog) [24] to promote megakaryocytic development [25]. However, these changes in gene expression are not accompanied by a simultaneous increase either in the expression of GP Ibα and GP IX, two GP Ib-IX-V subunits that only appear in the late stage of megakaryocyte differentiation; or in the expression of *NF-E2* (nuclear factor, erythroid-derived 2), a transcriptional factor that is important for the late stage of megakaryocyte differentiation [26]. These observations suggest that *HoxB4* may play a role in the early stage of megakaryocyte development. In agreement, it has been reported that the expression levels of miR-10a were significantly down-regulated by ~50-fold [15], which occurred 4 days after in vitro megakaryocytic differentiation of hematopoietic CD34^+^ progenitor cells. In a similar in vitro CD34^+^ differentiation system, inversely, GP Ibα mRNA levels were found to be drastically increased within the same time period [3]. Based on these previous observations and the data from this study, it is conceivable that active expression of human GP Ibα in the late stage of megakaryocyte differentiation depends on a significant reduction of the miR-10a expression. Nevertheless, several interesting questions remain to be answered. One, how is the GP Ibα mRNA degraded by miR-10a in megakaryocytes? How is the miR-10a host gene, *HoxB4*, regulated during megakaryocyte differentiation? Does dysregulated expression of *HoxB4* alter normal megakaryopoiesis due to a simultaneous dysregulation of miR-10a production and therein *GP Ibα* expression? Furthermore, although there has been no report in the literature identifying a role for miR-10b and its host gene, *HoxD4* (Homeobox D4), in megakaryopoiesis, Garzon et al. reported that in the late stage of megakaryopoiesis this miRNA is significantly down-regulated by ~12-fold [15]. Based on their observation and the data from this study, we postulate that miR-10b and its host gene, *HoxD4*, have unrealized roles in megakaryopoiesis, a hypothesis that awaits further investigations.

Our study also identified miR-107 and miR-299-3p as being responsible for an up-regulation in the expression of human GP Ibα mRNA. Even though we do not know the exact mechanism at this point, recent publications have revealed that miRNAs can act as activators to up-regulate gene expression, likely through enhancing mRNA stability as well as translational mechanisms [27,28]. Of these two miRNAs, interestingly, miR-299-3p is predicted to bind the 3′-UTRs of both human and mouse *GP1BA* gene and thus is potentially able to play important roles in GP Ibα expression and megakaryopoiesis in both human and mouse, a speculation worthy of further investigations. Meanwhile, because the miRNA-based regulatory mechanism for GP Ib expression is a largely unexplored area, our study does not exclude the possibility that there are miRNA species that can bind to the 5′-UTR and the coding sequence of the human *GP1BA* gene to also regulate gene expression. In our study, we searched for potential binding sites for miR-10a and -10b in these two regions of the human *GP1BA* gene, and found that only the 3′-UTR possesses such sites. Meanwhile, because miR-10a and -10b do not introduce any mutations to the GP Ibα coding sequence, and thereby do not alter the amino acid composition in the mature GP Ibα protein, it is unlikely that miR-10a and miR-10b can alter GP Ib-IX-V complex formation. However, sustaining high levels of miR-10a and miR-10b cause degradation of GP Ibα mRNA, resulting in low amounts of GP Ibα protein. This culminates in decreased levels of expression of the GP Ib-IX-V complex on the surface of platelets which may eventually inhibit human megakaryopoiesis and platelet production (Figure 3).

Taken together, our study identifies GP Ibα as a novel and physiologically relevant target of miR-10a and -10b, and provides a new piece of information to our pool of knowledge regarding the miRNA-based regulation of megakaryopoiesis. In particular, we have established a connection between two previous observations, i.e., drastic decrease in the expression of miR-10a and -10b accompanied by a marked production of sufficient amounts of GP Ibα molecules in the late stage of megakaryocyte development, two critical steps for normal megakaryocytic endomitosis and platelet production. In addition, our study provides a platform for future investigations seeking to identify novel inhibitory miRNA species responsible for the regulation of platelet-specific gene expression.

## 4. Materials and Methods

### 4.1. Prediction of Putative miRNA Binding Sites in the 3′-UTR of Human GP1BA Gene

Three web-based miRNA target prediction tools, TargetScan [29] (Whitehead Institute for Biomedical Research, Cambridge, MA, USA), miRanda [30] (Memorial Sloan-Kettering Cancer Center, New York City, NY, USA) and PITA [31] (Segal Lab of Computational Biology, Weizmann Institute, Rehovot, Israel) were used to analyze the 3′-UTRs of all four human GP Ib genes (*GP1BA*, *GP1BB*, *GPIX* and *GPV*).

### 4.2. Generation of GP1BA 3′-UTR Reporter Constructs and Site-Directed Mutagenesis

A 466 base pair region of the human *GP1BA* 3′-UTR was amplified from the pDX-hGP Ibα plasmid, a construct that contains both coding and 3′-UTR sequences of the human *GP1BA* gene [32,33]. This region was then cloned into SacI and XbaI sites of the pmirGLO vector (Promega, Madison, WI, USA), at an insertion site which is downstream of the stop codon of a firefly luciferase reporter gene (WT-3′-UTR reporter). Specific primers used in the polymerase chain reaction (PCR) were ATTGAGCTCGGGTGGGAGGTTTGGGG (forward) and CGGTCTAGACCCAACCCTAAAATTATTTTTTATTATACAGATAATATACAATAATAGTGG (reverse). Site-directed mutagenesis was performed to remove the putative miR-10a or -10b recognition sites in these constructs (MT-3′-UTR reporter) by using the following two primers (Stratagene): 5′-CCCTCCCTATCAGGGAGTGTTCCTTACCTCCAAC-3′ and 5′-AGGAACACTCCCTGATAGGGAGGGGTCTTAGTTCC-3′.

### 4.3. Cell Lines

Chinese Hamster Ovary (CHO) cells expressing human GP Ibβ and GP IX were cultured with an α-minimal essential medium supplemented with 10% heat-inactivated fetal bovine serum (FBS), 2 mM l-glutamine, 80 µg/mL methotrexate, 400 µg/mL G418, 400 µg/mL hygromycin, 100 U/mL penicillin, and 100 U/mL streptomycin in a humidified incubator containing 5% CO_2_ at 37 °C [33]. HeLa cells were cultured at 37 ° C with 5% CO_2_ in Dulbecco′s Modified Eagle′s Medium containing 10% heat-inactivated fetal bovine serum (FBS), 2 mM l-glutamine, 100 U/mL penicillin, and 100 U/mL streptomycin.

### 4.4. Luciferase Assays

HeLa cells were seeded in a 96-well plate (4 × 10^4^ cells per well). One day after seeding, the cells were transfected with 0.2 µg WT- or MT-3′-UTR reporter plasmid DNAs along with various concentrations of miRNA or negative control miRNA mimics (Qiagen, Valencia, CA, USA) using an Attractene Transfection Reagent (Qiagen). 40 h after transfection, the firefly and renilla luciferase activities in the cell lysates (Dual Luciferase Assay Kit, Promega) were measured by a 96 microplate luminometer. Each experiment was repeated at least three times in triplicate (*n* = 3). The luciferase activities are presented as mean ± SEM.

### 4.5. Flow Cytometry Analysis for Transient Expression of GP Ibα in GP Ibβ/IX-Expressing Chinese Hamster Ovary (CHO) Cells

Human GP Ibβ/IX-expressing CHO cells were seeded in a 24-well plate at a density of 1 × 10^5^ cells per well. One day after seeding, the cells were transfected with 0.5 μg pDX-hGP Ibα plasmid DNAs along with 80 nM of miR-10a/b or negative control miRNA mimics using an Attractene Transfection Reagent. 48 h after transfection, the cells were stained with a mouse anti-human GP Ibα specific antibody, SZ2 (Beckman Coulter, Brea, CA, USA), followed by labeling with a fluorescein isothiocyanate (FITC)-conjugated anti-mouse secondary antibody (Invitrogen, Carlsbad, CA, USA). The GP Ibα expression levels were then measured with a Coulter Epics XL-MCL Flow Cytometer and analyzed using EXPO32 ADC software version 1.2 (Beckman Coulter, Brea, CA, USA). Mean fluorescence intensities were normalized to that of the cells treated with negative control miRNA mimics. Standard errors (±SEM) were calculated from three independent experiments.

### 4.6. In Vitro Culture and Transfection of Murine Lineage Negative Cells

Total bone marrow cells were collected from the femurs of mice whose genome is integrated with a ~6-kb EcoRI fragment possessing an entire cassette of the human *GP1BA* gene (including native human *GP1BA* 3′-UTR) to induce a high level of human GP Ibα expression in murine platelets and megakaryocytes [20,21]. After lysis of red blood cells with a red blood cell (RBC) lysis buffer, the lineage negative cells (Lin^−/−^) were enriched with a BD IMag™ Mouse Hematopoietic Progenitor Cell Enrichment Set Kit according to the manufacturer′s instructions (BD Bioscience, San Jose, CA, USA). A half million enriched Lin^−/−^ cells were first transfected with 80 nM of miR-10a/b mimics or inhibitors (Qiagen) or negative control miRNA mimics using a HiPerFect Transfection Reagent (Qiagen). The transfected cells (1 × 10^6^ cells/mL) were then cultured in a Iscove′s Modified Dulbecco′s Medium (IMDM, Invitrogen) supplemented with 50 ng/mL recombinant mouse thrombopoietin, 10 ng/mL recombinant mouse interleukin-3 (IL-3) and 20 ng/mL recombinant human IL-6 (BD Bioscience) to allow megakaryocyte differentiation for 4 days. The use of all animals involved in these experiments have been carried out in accordance with the Animal Welfare Act, PHS Animal Welfare Policy; the principles of the NIH Guide for the Care and Use of Laboratory Animals, and the policies and procedures of Baylor College of Medicine. The animal experiments in this project were approved by the Institutional Animal Care and Use Committee of Baylor College of Medicine (animal protocol #AN-4413, 5 Aug 2015).

### 4.7. Reverse Transcription Polymerase Chain Reaction (RT-PCR)

Total RNA was isolated using a Qiagen RNeasy mini kit at 0, 2, and 4 days after megakaryocytic induction. One microgram of the total RNA and a random primer mixture provided by Promega were used for the reverse transcription reaction. Subsequently, one microgram of the yielded cDNA was used as a PCR template to detect human GP Ibα mRNA expression, in which two human GP Ibα specific primers were used to amplify a 455 nt human *GP1BA* gene fragment: 5′-GAGAGAAGGACGGAGTCGAGTGGC-3′ (forward, in exon 1) and 5′-CGGTTGAAGGAGACGTCCAGGACG-3′ (reverse, in exon 2). As a control, a 238 nt of mouse GAPDH gene fragment was also amplified with the following two primers [34]: 5′-CTTCACCACCATGGAGAAGGC-3′ (forward) and 5′-GGCATGGACTGTGGTCAT-3′ (reverse). Ten microliters of each reaction mixture were then run in a 2% agarose gel and stained with ethidium bromide. The expression levels of human GP Ibα mRNA were analyzed by densitometry using Image J software.

### 4.8. Quantitative Polymerase Chain Reaction (qPCR)

RNA was isolated from harvested cells with the RNAeasy Micro Kit (Qiagen), and subsequent reverse transcription was carried out by using a miScript Reverse Transcription Kit (Qiagen). SYBR green-based qPCR was performed on an ABI PRISM Sequence Detection System and the level of gene expression was normalized to GAPDH. The primers for human GP Ibα were 5′-AGGTCTTTCTGCCTGCCTGTC-3′ (forward) and 5′-GGCGAGTGTAAGGCATCAGG-3′ (reverse). The primers for mouse GAPDH were 5′-TCGTCCCGTAGACAAAATGG-3′ (forward) and 5′-TTGAGGTCAATGAAGGGGTC-3′ (reverse). PCR conditions for GP Ibα were as follows: 95 °C for 15 min followed by 40 cycles of 94 °C for 15 s, 60 °C for 30 s and 70 °C for 40 s. To quantify miR-10a and -10b, miRNA specific primers and miScript Universal primers were purchased from Qiagen and relative mature miRNA levels were normalized to U6. PCR conditions for miR-10a and miR-10b were as follows: 95 °C for 15 min followed by 40 cycles of 94 °C for 15 s, 57 °C for 30 s and 70 °C for 40 s. The 2^−ΔΔ*C*t^ method was used to determine relative expression levels.

### 4.9. Statistical Analysis

Data were expressed as mean ± SEM. The Student *t* test and analysis of variance were used to assess differences. *p* < 0.05 is considered significant.

## Abbreviations

3′-UTR	Three prime untranslated region
BSS	Bernard-Soulier Syndrome
GP	Glycoprotein
vWf	Von Willebrand factor
FBS	Fetal bovine serum
RT-PCR	Reverse Transcription Polymerase Chain Reaction
qPCR	Quantitative Polymerase Chain Reaction
CHO	Chinese Hamster Ovary
IMDM	Iscove′s Modified Dulbecco′s Medium
FITC	Fluorescein isothiocyanate
RBC	Red blood cell
GATA	GATA binding protein
Ets	E26 transformation-specific sequence family transcriptional factor
Meis1	Meis homeobox 1
MAFB	V-maf musculoaponeurotic fibrosarcoma oncogene homolog B
c-Myb	Avian myeloblastosis virus oncogene cellular homolog
NF-E2	Nuclear factor, erythroid-derived 2
HoxB4	Homeobox B4
HoxD4	Homeobox D4
